# Fungal Burden and Raised Intracranial Pressure Are Independently Associated With Visual Loss in Human Immunodeficiency Virus-Associated Cryptococcal Meningitis

**DOI:** 10.1093/ofid/ofab066

**Published:** 2021-02-05

**Authors:** Síle F Molloy, Brad Ross, Cecilia Kanyama, Sayoki Mfinanga, Sokoine Lesikari, Robert S Heyderman, Newton Kalata, Jayne Ellis, Charles Kouanfack, Duncan Chanda, Elvis Temfack, Shabir Lakhi, Anand Moodley, Adrienne K Chan, Joep J van Oosterhout, Yacouba Mapoure, Peter Mwaba, David G Lalloo, Duolao Wang, Olivier Lortholary, Shabbar Jaffar, Mina C Hosseinipour, Angela Loyse, Thomas S Harrison, Tihana Bicanic

**Affiliations:** 1 Centre for Global Health, Institute of Infection and Immunity, St George’s University of London, London, United Kingdom; 2 Clinical Academic Group in Infection, St George’s University Hospital, LondonUnited Kingdom; 3 UNC Project, Kamuzu Central Hospital, Lilongwe, Malawi; 4 National Institute for Medical Research, Muhimbili Centre, Dar es Salaam, Tanzania; 5 Liverpool School of Tropical Medicine, Liverpool, United Kingdom; 6 University College London, London, United Kingdom; 7 Malawi-Liverpool-Wellcome Trust Clinical Research Programme, Blantyre, Malawi; 8 College of Medicine, Queen Elizabeth Hospital, Blantyre, Malawi; 9 The Hospital for Tropical Diseases, University College Hospital, LondonUnited Kingdom; 10 Hȏpital Central Yaoundé/Site ANRS Cameroun, Yaoundé, Cameroon; 11 Institute for Medical Research and Training, University Teaching Hospital, Lusaka, Zambia; 12 Douala General Hospital, Douala, Cameroon; 13 National Public Health Laboratory, Yaounde, Cameroon; 14 University Teaching Hospital, Lusaka, Zambia; 15 Department of Neurology, University of the Free State, Bloemfontein, South Africa; 16 Dignitas International, Zomba Central Hospital, Zomba, Malawi; 17 Division of Infectious Diseases, Department of Medicine, Sunnybrook Health Sciences Centre, University of Toronto, Toronto, Canada; 18 Partners in Hope, LilongweMalawi; 19 David Geffen School of Medicine, University of California, Los Angeles, USA; 20 Department of Internal Medicine and Directorate of Research and Post Graduate Studies, Lusaka Apex Medical University, Lusaka, Zambia; 21 Institut Pasteur, Molecular Mycology Unit, Centre National de la Recherche Scientifique, Paris, France; 22 Paris Descartes University, Necker Pasteur Center for Infectious Diseases and Tropical Medicine, Institut Imagine, Assistance Publique – Hôpitaux de Paris, Paris, France; 23 University of North Carolina, Chapel Hill, North Carolina, USA

**Keywords:** cryptococcal meningitis, fungal burden, HIV, raised intracranial pressure, visual loss

## Abstract

Among 472 patients with human immunodeficiency virus-associated cryptococcal meningitis, 16% had severe visual loss at presentation, and 46% of these were 4-week survivors and remained severely impaired. Baseline cerebrospinal fluid opening pressure ≥40 cmH_2_O (adjusted odds ratio [aOR], 2.56; 95% confidence interval [CI], 1.36–4.83; *P* = .02) and fungal burden >6.0 log_10_ colonies/mL (aOR, 3.01; 95% CI, 1.58–5.7; *P* = .003) were independently associated with severe visual loss.

Human immunodeficiency virus (HIV)-associated cryptococcal meningitis (CM) is the most common cause of adult meningitis in sub-Saharan Africa, and it accounts for ~180 000 deaths/year globally [1]. In addition to headache, CM often presents with altered mental status, raised intracranial pressure (ICP), and hearing and visual loss [2], with survivors often experiencing neurological sequelae [3–5]. In CM, visual loss is usually bilateral, can be sudden or gradual in onset, occurs before or during antifungal treatment, and—especially without intervention—is frequently irreversible [3, 5, 6]. Evaluation of visual acuity in encephalopathic patients is challenging: the few CM cohorts (HIV and non-HIV) that systematically examined visual acuity report a prevalence of 33%–46% for any visual impairment (<6/6 Snellen chart), with profound visual loss (<6/60) occurring in 13% of patients [5–7].

Proposed mechanisms of visual loss include direct optic nerve infiltration by cryptococci (demonstrated on autopsy as well as tissue biopsy), inflammatory arachnoiditis, or optic nerve compression through raised ICP or a more localized optic nerve compartment syndrome [5], with both fungal burden and raised ICP implicated in its pathophysiology [3, 8]. Prior, small retrospective cohorts (<100 patients) suggested that high fungal burden (cerebrospinal fluid [CSF] cryptococcal antigen titer >1024) or raised ICP (CSF opening pressure [OP] >30 cmH_2_O) are associated with visual loss in CM, but they have been too small to undertake multivariable analyses [3, 6]. In this study, we report on the prevalence, risk factors, and reversibility of visual loss in a large prospective CM patient multicountry cohort in sub-Saharan Africa [9].

## METHODS

Anonymized data from the Advancing Cryptococcal Meningitis Treatment in Africa ([ACTA] ISCRTN 45035509) trial formed the dataset for this preplanned substudy [9]. Between 2013 and 2016, 678 adults with HIV CM were randomized to receive oral fluconazole plus flucytosine or amphotericin B-based therapy for 1 or 2 weeks. Patients had protocol-specified lumbar punctures (LPs) on day 1, 7, and 14 with OP measurements and therapeutic CSF drainage according to guidelines [10].

Visual acuity (VA) was measured at baseline (≤3 days from enrollment) and at 4 weeks using the standardized logMAR chart in surviving patients whose conscious level permitted assessment. The score, ranging from 0 (able to read all letters on the smallest line) to 1.375 (unable to read any letters), was recorded for each eye. Those unable to read any letters were assessed for finger counting, hand movement, and light perception. Those without light perception were considered blind. Patients were classified into 6 categories: (1) near-normal vision (score <0.5), (2) moderate visual loss (score ≥0.5 but <1.0), (3) finger counting (score ≥1.0 and able to count fingers), (4) hand motion perception, (5) light perception, or (6) no light perception. When VA varied between the left and right eye (9.3%, 44 of 472), data for the worst eye were analyzed. Patients did not routinely undergo computed tomography brain or fundoscopy to assess for papilloedema or other ocular pathology.

Data were analyzed using Stata v15 (StataCorp, College Station, Texas, United States), using Kruskal-Wallis tests for continuous variables, chi-squared tests for categorical variables and quantile regression to test for trend across the 6 visual acuity categories. Based on proposed pathophysiological mechanisms, univariable and multivariable logistic regression was used to investigate the association between baseline visual loss and CSF white cell count (WCC), OP, and fungal burden, adjusted a priori for age. Sex, CD4 count, and antiretroviral therapy status (naive or exposed) were not associated with visual loss, and no other measured variables were hypothesized to confound the relationship. For analysis of the association among VA, CSF, OP, and fungal burden, VA was dichotomized into near-normal/moderate vision (categories 1 and 2) and severe visual loss (categories 3–6), similar to definitions in [4].

To assess impact of antifungal therapy and therapeutic LPs on reversibility of visual impairment, rate of clearance of infection, number of LPs performed, total CSF volume removed, and change in OP over 2 weeks’ treatment was compared (1) between those with repeat measurements at 4 weeks in whom acuity deteriorated and those who improved and (2) in those that remained severe versus improved from severe.

### Patient Consent Statement

Written informed consent was obtained from all participants in ACTA. Ethical approval was granted by the London School of Hygiene and Tropical Medicine Research Ethics Committee and by national research ethics committees and regulatory bodies in Malawi, Zambia, Cameroon, and Tanzania.

## RESULTS

### Baseline Data

Of 678 ACTA participants, VA was measured in 472 (69.6%) at baseline. Seventy-five patients (15.9%) had severe visual loss with 20 (4%) classed as blind. One fifth (15 of 75) of those with severe visual loss also reported hearing loss.

Overall, 206 (47.1%) patients had raised baseline ICP (OP ≥25 cmH_2_O) and median fungal burden was 4.9 log_10_ colonies/mL (interquartile range, 3.7–5.8) (Supplementary Table 1). Cryptococcus *neoformans* was the predominant species causing CM in this African cohort (337 of 382 sequenced isolates). Due to difficulties in measuring VA in unconscious patients, the VA cohort had less abnormal mental status (Glasgow Coma Scale <15) (12.5% vs 50.5%, *P* < .001) and lower median OP (19 vs 25 cmH_2_O, *P* < .001), but no significant differences in median fungal burden (4.9 vs 5.1 log_10_ colonies/mL, *P* = .74), compared with the entire trial cohort.

### Association Among Severe Baseline Visual Loss, Opening Pressure, and Fungal Burden

**Figure 1.**
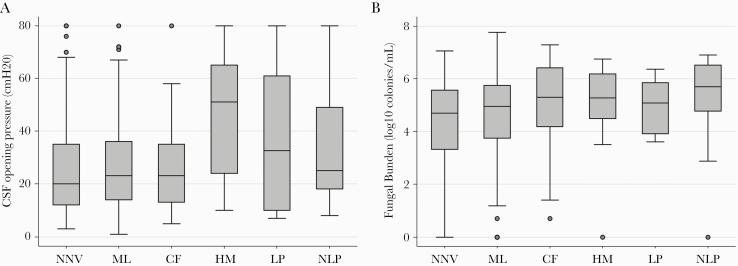
(a) Box plot (median, interquartile range, range) of baseline cerebrospinal fluid (CSF) opening pressure by visual acuity group at presentation. (b) Box plot of baseline fungal burden by visual acuity group at presentation. CF, count fingers; HM, hand motion; LP, light perception; ML, moderate loss; NN, near normal; NNV, near-normal vision; NV, no vision (blind); PL, perceives light.

Across 6 categories from near-normal to blind, decreasing VA was associated with both increased OP and higher fungal burden at baseline (*P* = .02 and *P* < .008, respectively) (Figure 1a and b). High OP and fungal burdens were also associated, with 55 (25.6%) patients with fungal burden above the median (≥4.9 log_10_ colonies/mL) also experiencing high OP (>40 cmH_2_O), compared with 33 (15.4%) with lower fungal burden (*P* = .009).

In multivariable analyses, age, baseline CSF pressure (<25 cm vs ≥40 cmH_2_O [adjusted odds ratio {aOR}, 2.56; 95% confidence interval {CI}, 1.36–4.83; *P* = .02]), and fungal burden ([<5 vs >6.0 log_10_ colonies/mL] aOR, 3.01; 95% CI, 1.58–5.7; *P* = .003) were independently associated with severe visual loss (Table 1). There was no association between baseline central nervous system inflammation (CSF WCC >10 × 10^9^/L) and visual loss on either univariable or multivariable analyses.

**Table 1.** Univariable and Multivariable Analysis for the Association Between Age, Baseline CSF Opening Pressure, Baseline CSF Fungal Burden, and CSF White Cell Count and Severe Visual Loss at Presentation

	Visual Acuity at CM Presentation, no. (%)					
Variable (Baseline)	Normal or Moderate Loss	Severe Loss	Unadjusted OR (95% CI)	*P* Value	Adjusted OR (95% CI)	*P* Value*
Age						
<50 year	356 (89.7)	41 (10.3)	1		1	
≥50 years	61 (81.3)	14 (18.7)	1.99 (1.03–3.87)	.04	2.47 (1.22–5.05)	.04
CSF Opening Pressure (cmH_2_O)						
<25	200 (86.6.0)	31 (13.4)	1		1	
25–39	98 (85.2)	17 (14.8)	1.12 (0.59–2.12)		1.28 (0.66–2.47)	
≥40	66 (72.5)	25 (27.5)	2.44 (1.35–4.43)	.003	2.56 (1.36–4.83)	.02
Fungal Burden (log_10_ colonies/mL)						
<5.0	206 (88.4)	27 (11.6)	1		1	
5.0–5.9	113 (84.3)	21 (15.7)	1.42 (0.77–2.62)		1.31 (0.69–2.47)	
>6.0	62 (70.5)	26 (29.5)	3.20 (1.74–5.88)	.001	3.01 (1.58–5.70)	.003
White Cell Count (×10^9^/L)						
<10	249 (82.2)	54 (17.8)	1		1	
≥10	126 (86.3)	20 (13.7)	0.73 (0.42–1.28)	.27	0.70 (0.39–1.25)	.22

Abbreviations: CI, confidence interval; CM, cryptococcal meningitis; CSF, cerebrospinal fluid; OR, odds ratio.

*Adjusted for each variable in the table.

### Changes in Visual Acuity Over Time

Paired VA data were available for 336 of 472 (71.2%) patients at baseline and week 4. Of these, 171 (50.9%) had normal vision, 128 (38.1%) had moderate vision, and 37 (11.0%) had severe visual loss at baseline. Death before follow-up was the main reason for missing data at 4 weeks (98 of 136, 72.1%).

Visual acuity remained unchanged over time for 210 (62.5%) patients. No significant differences were observed in rate of clearance of infection, number of LPs performed, total volume of CSF removed, and change in OP over the first 2 weeks of treatment between the group remaining the same, those in whom VA deteriorated (n = 51, 15.2%), and those that improved (n = 75, 22.3%) (*P* > .1, all comparisons) (Supplementary Table 2).

Of 37 (11%) patients with severe visual loss at baseline, 12 (32.4%) improved to near-normal, 8 (21.6%) improved to moderate, and 17 (46.0%) remained severe. Again, there were no significant differences in the above parameters between patients remaining severely impaired and those improving (Supplementary Table 3), although numbers for comparison were low.

## DISCUSSION

This prospective study within a large African trial is the first to demonstrate an independent association of both fungal burden and raised ICP, in addition to age, with visual loss at presentation with HIV-CM. At presentation, 16% of patients had severe visual loss and 4% were blind: in those with repeat measurements at 4 weeks, after fungicidal treatment regimens and aggressive management of raised ICP, 54% of patients with severe baseline visual loss improved to near-normal or moderate vision, whereas 46% remained severely impaired.

In a case series comprising both HIV-infected and uninfected patients (n = 49), the authors proposed dichotomous mechanisms of visual loss, optic neuritis due to fungal infiltration accounting for early rapid visual loss, and optic nerve compression due to raised ICP accounting for a slower onset. Interventions aimed at reducing ICP were the only ones associated with any success [3]. In a study of immunocompetent adults with *Cryptococcus gattii* meningitis, in which ICP was not consistently measured or managed, 37% of 57 survivors remained blind, compared with 5% in our cohort [6]. The uniformity of our cohort, in terms of use of fungicidal regimens and pressure management, may not have permitted us to show a significant impact of rate of clearance or changes in pressure-related parameters on reversibility of visual loss.

In the most comprehensive mechanistic study of visual loss in HIV-CM [5] researchers observed frequent optic nerve conduction and visual field defects compatible with raised ICP and no evidence of optic neuritis on MRI. The authors present evidence for an optic nerve compartment syndrome, caused by cryptococcal plugging of channels between the intracranial and the peri-optic subarachnoid space, as an additional cause of optic nerve dysfunction in CM [8]. In practice, these mechanisms may well overlap [8]: our prior work [2] and findings from this study suggest a relationship between fungal burden and raised pressure at the highest extremes of each. These 2 factors likely converge in an individual patient, possibly more susceptible due to neuroanatomical variation, rendering them either temporarily or—in the absence of intervention—permanently visually impaired.

## CONCLUSIONS

Limitations of our cohort include the lack of visual assessment at baseline or reversibility at 4 weeks in the sickest of patients who subsequently die, possibly underestimating the prevalence of visual loss. We did not capture self-reported visual impairment predating CM onset, which may have impacted on baseline VA, nor were we able to exclude other conditions that can impair vision in this advanced HIV cohort (eg, cytomegalovirus retinitis). Nonetheless, our findings underscore the importance of interventions targeted at both fungal burden and raised ICP in mitigating visual loss, and they underline the need for continued efforts towards earlier diagnosis and treatment of CM, given that approximately half of those with severe impairment failed to improve despite fungicidal regimens and aggressive management of raised CSF pressure.

## Supplementary Data

Supplementary materials are available at Open Forum Infectious Diseases online. Consisting of data provided by the authors to benefit the reader, the posted materials are not copyedited and are the sole responsibility of the authors, so questions or comments should be addressed to the corresponding author.

ofab066_suppl_Supplementary_Tables

## References

[1] Rajasingham R , Smith RM, Park BJ, et al Global burden of disease of HIV-associated cryptococcal meningitis: an updated analysis. Lancet Infect Dis 2017; 17:873–81. 28483415 10.1016/S1473-3099(17)30243-8 PMC5818156

[2] Bicanic T , Brouwer AE, Meintjes G, et al Relationship of cerebrospinal fluid pressure, fungal burden and outcome in patients with cryptococcal meningitis undergoing serial lumbar punctures. AIDS 2009; 23:701–6. 19279443 10.1097/QAD.0b013e32832605fe

[3] Rex JH , Larsen RA, Dismukes WE, et al Catastrophic visual loss due to *Cryptococcus neoformans* meningitis. Medicine (Baltimore) 1993; 72:207–24. 8341139 10.1097/00005792-199307000-00001

[4] Loyse A , Moodley A, Rich P, et al. Neurological, visual, and MRI brain scan findings in 87 South African patients with HIV-associated cryptococcal meningoencephalitis. J Infect 2015; 70:668–75. 10.1016/j.jinf.2014.10.007 25444972

[5] Moodley A , Rae W, Bhigjee A, et al Early clinical and subclinical visual evoked potential and Humphrey’s visual field defects in cryptococcal meningitis. PLoS One 2012; 7:e52895. 23285220 10.1371/journal.pone.0052895 PMC3528708

[6] Seaton RA , Verma N, Naraqi S, et al Visual loss in immunocompetent patients with *Cryptococcus neoformans* var. *gattii* meningitis. Trans R Soc Trop Med Hyg 1997; 91:44–9. 9093627 10.1016/s0035-9203(97)90391-6

[7] Jarvis JN , Bicanic T, Loyse A, et al Determinants of mortality in a combined cohort of 501 patients with HIV-associated cryptococcal meningitis: implications for improving outcomes. Clin Infect Dis 2014; 58:736–45. 24319084 10.1093/cid/cit794 PMC3922213

[8] Moodley A , Rae W, Bhigjee A. Visual loss in HIV-associated cryptococcal meningitis: a case series and review of the mechanisms involved. South Afr J HIV Med 2015; 16:305. 29568574 10.4102/sajhivmed.v16i1.305 PMC5843184

[9] Molloy SF , Kanyama C, Heyderman RS, et al; ACTA Trial Study Team. Antifungal combinations for treatment of cryptococcal meningitis in Africa. N Engl J Med 2018; 378:1004–17. 29539274 10.1056/NEJMoa1710922

[10] Perfect JR , Dismukes WE, Dromer F, et al. Clinical Practice Guidelines for the Management of Cryptococcal Disease: 2010 update by the Infectious Disease Society of America. Clin Infect Dis 2010; 50:291–322. 20047480 10.1086/649858 PMC5826644

