# Supplementary material for: Fungal Burden and Raised Intracranial Pressure Are Independently Associated With Visual Loss in Human Immunodeficiency Virus-Associated Cryptococcal Meningitis
Source: Open Forum Infect Dis. 2021 Feb 5;8(4):ofab066. doi: 10.1093/ofid/ofab066 (PMC8078267; doi:10.1093/ofid/ofab066)
Supplement: ofab066_suppl_Supplementary_Tables [file ofab066_suppl_supplementary_tables.docx]

**Supplementary Table 1**: Baseline characteristics

| **Characteristic** | **N** | **no. (%) or**  **median (IQR)** | |
| --- | --- | --- | --- |
| **Age** (years) | 472 | 38 | (31 - 44) |
| **Sex** (male) | 472 | 267 | (56.6) |
| **Visual Acuity of Worse Eye –** no. (%)   - Near-Normal - Moderate Loss - Count Fingers - Hand motion - Light perception - No light perception (blind) | 472 | 220  177  30  17  8  20 | (46.6)  (37.5)  (6.4)  (3.6)  (1.7)  (4.2) |
| **Severe Visual Loss** | 472 | 75 | (15.9) |
| **VI Cranial Nerve Palsy** | 472 | 22 | (4.7) |
| **Hearing Loss** | 472 | 34 | (7.4) |
| **Severe visual loss and hearing loss** | 472 | 15 | (3.2) |
| **Abnormal Mental Status*** | 472 | 59 | (12.5) |
| **Fungal burden** (log_10_ colonies/ml) | 455 | 4.9 | (3.7 – 5.8) |
| **CSF opening pressure^+^** (cm H_2_0) | 437 | 22 | (13 – 36) |
| **Raised CSF opening pressure**  (≥25cm H_2_0) | 437 | 206 | (47.1) |
| **CSF White cell count**  (≥10 x 10^9^/L) | 449 | 146 | (32.5) |

*Abnormal mental status indicates a Glasgow Coma Scale (GCS) score <15

**^+^** maximum recorded 80 cm (using 2 stacked manometers)
Abbreviations: CSF (Cerebrospinal Fluid)

**Supplementary Table 2**: Comparison of parameters for patients with improved or unchanged visual acuity with those that deteriorated from baseline to 4 weeks

|  | Change in visual acuity status over time* | | |
| --- | --- | --- | --- |
|  | **Remained the same (n=210)** | **Improved from baseline**  **(n=75)** | **Deteriorated from baseline**  **(n=51)** |
| Rate of clearance of infection (mean, SD) | 0.38 (0.25) | 0.38 (0.25) | 0.36 (0.21) |
| Change in OP from baseline to Day 14 (cm H2O) (mean, SD) | -5 (20.7) | -6 (15.6) | -7 (14.2) |
| Number of LPs  (median, IQR) | 3 (3-5) | 3 (3-5) | 3 (3-4) |
| Volume of CSF removed (mls)  (median, IQR) | 35 (20-60) | 35 (20-58) | 32 (20-50) |

*p-values for ANOVA tests for difference in means and Quantile regression for difference in medians were non-significant (p>0.1 for all group comparisons), with deteriorated from baseline as the reference category

**Supplementary Table 3**: Comparison of parameters for patients that remained with severe visual loss from baseline to 4 weeks with those that improved

|  | Change in visual acuity status over time | | |
| --- | --- | --- | --- |
|  | **Remained severe**  **(n=17)** | **Improved from severe (n=20)** | **p-value** |
| Rate of clearance of infection (mean, SD) | 0.36 (0.2) | 0.35 (0.26) | 0.88* |
| Change in OP from baseline to Day 14 (cm H2O) (mean, SD) | -6.5 (20) | -12.6 (15.4) | 0.35* |
| Number of LPs (median, IQR) | 3 (3-5) | 4 (3-6) | 0.31** |
| Volume of CSF removed (mls) (median, IQR) | 50 (21-62) | 36 (17-57) | 0.29** |

*t-test **Kruskal-Wallis test
